# Highly deregulated lncRNA LOC is associated with overall worse prognosis in Hepatocellular Carcinoma patients

**DOI:** 10.7150/jca.56340

**Published:** 2021-03-30

**Authors:** Lee Jin Lim, Lay Hiang Ling, Yu Pei Neo, Alexander Y.F. Chung, Brian K.P. Goh, Pierce K.H. Chow, Chung Yip Chan, Peng Chung Cheow, Ser Yee Lee, Tony K.H. Lim, Samuel S. Chong, London L. P. J. Ooi, Caroline G. Lee

**Affiliations:** 1 Dept of Biochemistry, Yong Loo Lin School of Medicine, National University of Singapore, Singapore.; 2 Dept of Hepato-pancreato-biliary & Transplant Surgery, Singapore General Hospital, Singapore.; 3 Duke-NUS Medical School, Singapore.; 4 Dept of Surgical Oncology, National Cancer Centre Singapore, Singapore.; 5 Dept of Pathology, Singapore General Hospital, Singapore.; 6 Div of Cellular & Molecular Research, Humphrey Oei Institute of Cancer Research, National Cancer Centre Singapore, Singapore.; 7 Department of Pediatrics, Yong Loo Lin School of Medicine, National University of Singapore, Singapore.; 8 NUS Graduate School for Integrative Sciences and Engineering, National University of Singapore, Singapore.

**Keywords:** Hepatocellular Carcinoma, Long non-coding RNA, Prognosis, Hepatocarcinogenesis, LOC101926913

## Abstract

Although numerous long non-coding RNAs (lncRNAs) were reported to be deregulated in Hepatocellular Carcinoma (HCC), experimentally characterized, and/or associated with patient's clinical characteristics, there is, thus far, minimal concerted research strategy to identify deregulated lncRNAs that modulate prognosis of HCC patients. Here, we present a novel strategy where we identify lncRNAs, which are not only de-regulated in HCC patients, but are also associated with pertinent clinical characteristics, potentially contributing to the prognosis of HCC patients. LOC101926913 (LOC) was further characterized because it is the most highly differentially expressed amongst those that are associated with the most number of clinical features (tumor-stage, vascular and tumor invasion and poorer overall survival). Experimental gain- and loss-of-function manipulation of LOC in liver cell-lines highlight LOC as a potential onco-lncRNA promoting cell proliferation, anchorage independent growth and invasion. LOC expression in cells up-regulated genes involved in GTPase-activities and downregulated genes associated with cellular detoxification, oxygen- and drug-transport. Hence, LOC may represent a novel therapeutic target, modulating prognosis of HCC patients through up-regulating GTPase-activities and down-regulating detoxification, oxygen- and drug-transport. This strategy may thus be useful for the identification of clinically relevant lncRNAs as potential biomarkers/targets that modulate prognosis in other cancers as well.

## Introduction

Liver cancer is a particularly fatal form of cancer, being the sixth most frequently diagnosed and fourth most frequent cause of cancer mortality 1. Liver cancer comprises a diverse group of histologically distinct malignant tumors such as Hepatocellular Carcinoma (HCC), Intrahepatic Cholangiocarcinoma, Fibrolamellar Carcinoma and Hepatoblastoma 2, 3. HCC is the most common primary liver cancer, representing ~75-85% of liver cancer cases 1. Risk factors include chronic Hepatitis B and C virus infection, excessive alcohol intake, aflatoxin consumption, obesity, tobacco smoking and diabetes 1, 3. Although surgical resection and liver transplantation are potential curative treatments, the risk of recurrence within 5 years is still ~70% for HCC patients who undergo surgical resection and ~10-60% for liver transplantation 4-6. Furthermore, early stage of HCC is often asymptomatic, leading to late diagnosis of HCC and the treatment options for advanced HCC become essentially palliative 7-9. To date, HCC remains one of the few common cancers where there is no proven adjuvant therapy 10. Hence, it is important to identify and characterize molecules that modulate prognosis of HCC patients to facilitate the design of therapeutic targets to improve the outcome of HCC patients.

Early HCC studies primarily focus on protein-coding genes 11-14 since proteins are the key molecules that affect function 15. The advent of next-generation high-throughput sequencing technologies highlighted that protein coding genes only contribute <2% of the human transcriptome while >80% of the genome are transcribed as non-coding RNAs (ncRNAs) 15. Amongst the ncRNAs, long non-coding RNAs (lncRNAs), whose length are >200 bases, represent the largest class of ncRNAs comprising 68% of human transcriptome 16-19. Emerging evidence has shown that lncRNAs are important regulators in cellular processes modulating gene expression through various mechanisms 20, 21. Due to their key regulatory roles in the cells, their deregulation is often associated with carcinogenesis in various cancers including HCC 9, 22-24. While other studies have demonstrated that deregulated lncRNAs potentially regulate important network of genes in key cancer pathways 25-27, our laboratory has also reported that these deregulated lncRNAs in networks could act as potential master regulators in regulating patient prognosis 28.

**Figure 1:**
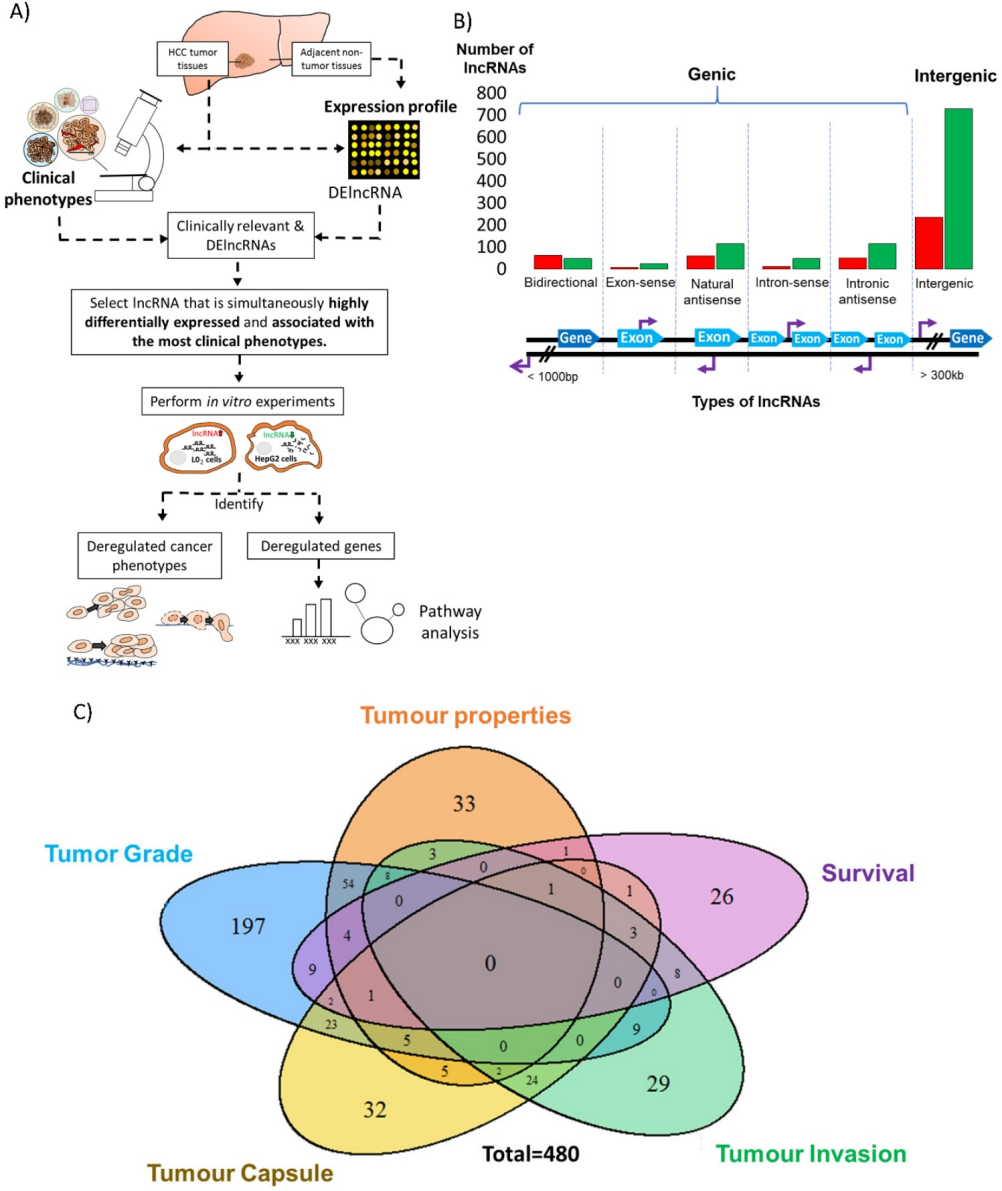
Identification of differentially expressed and clinically relevant lncRNAs. **A)** Schematic overview of the strategy to study differentially expressed and clinically relevant lncRNAs. LncRNA and mRNA expression profile were performed on HCC tumor tissues and adjacent non-tumorous tissues. Clinical phenotypes of HCC tumor tissues were used for clinical association studies. Differentially expressed (DE) and clinically relevant lncRNAs were then identified. Among the clinically relevant DElncRNAs, lncRNA that was simultaneously highly differentially expressed and associated with the most clinical phenotypes was selected for further analysis. Gain-and loss-of-function experiments in two cell lines were performed to study the cancer phenotypes and genes deregulated by the clinically relevant lncRNA. Pathway analysis was conducted on the deregulated genes. Colored circles: Five clinical phenotypes used in this study; Orange: Tumor properties; Yellow: Tumor capsule; Blue: Tumor grade; Green: Tumor invasion; Purple: Patient overall survival. **B)** Bar chart shows the number of differentially expressed lncRNAs that are grouped based on their relationship with protein-coding genes. The six types of lncRNAs are illustrated below the bar chart. Red: Upregulation in T vs NT; Green: Downregulation in T vs NT. Light blue thick arrows: Exons of protein coding genes; Dark blue thick arrows: Protein coding genes; Purple arrows: LncRNA transcription start site. **C)** Venn diagram shows the number of clinically relevant lncRNAs, which is defined as lncRNAs that is differentially expressed in T vs NT and also differentially expressed in poor clinical characteristic vs good clinical characteristic. Their expressions in both T vs NT and poor clinical characteristic vs good clinical characteristic are in the same direction and significant. Blue: Tumor Grade; Orange: Tumor properties (Includes tumor size, tumor stage and vascular invasion); Purple: Overall Survival; Green: Tumor invasion; Yellow: Tumor capsule (Includes Encapsulation and Degree of encapsulation).

Current HCC lncRNAs studies provides only fragmentary snapshots of lncRNAs role in modulating prognosis of patients as most primarily profile lncRNAs in HCC patients, characterize lncRNAs based on their expression levels or previous reports in other cancers and then determine if the characterized lncRNAs are associated with clinical characteristics 9. Here, we propose a novel strategy to identify lncRNAs that are pertinent for the prognoses of HCC patients (Figure 1A). Integrating the lncRNA expression profiles of the HCC patients with the clinical phenotype association in the same patients, we identified lncRNAs that are not only highly deregulated in the tumors of HCC patients, but are also simultaneously significantly associated with the most number of pertinent clinical phenotype in a consistent manner. Using this strategy, lncRNA LOC101926913 (Seqname: NR_110185) was identified to be a promising potential prognostic onco-lncRNA.

## Materials and methods

### Preparation of patient tissues samples

Patient tissues samples were collected and prepared as reported previously 28. Patients of either gender above the age of 21 with histologically confirmed HCC were included in this study, while pregnant women and vulnerable individuals as well as patients who did not consent to participate or were not diagnosed with HCC were excluded from this study. Tumor tissues and adjacent non-tumorous tissues were collected with informed consent from the patients at the Singapore General Hospital. This study was approved by SingHealth Institutional Review Board (SingHealth CIRB Ref: 2018/3155). All methods were carried out according to the World Medical Association Declaration of Helsinki.

### LncRNA and mRNA profiling of patient tissues and microarray data analysis

RNA extraction and preparation from tissue samples, lncRNA and mRNA profiling, as well as microarray data analysis were carried out as previously described 28.

### Clinically associated lncRNAs identification

Clinical association of lncRNAs was performed as described previously 28. Briefly, the clinical phenotypes were first grouped into five categories, which are tumor properties (tumor size, vascular invasion and tumor stage), tumor grade (Also known as Edmondson grade in HCC), tumor capsule (encapsulation and degree of encapsulation), tumor invasion and overall survival status (Figure S1). For each clinical phenotype, tumor samples were further divided into 'good characteristics' (which include tumor size <5 cm, absence of vascular invasion, lower stage (stage 1/2), lower tumor grade (grade 1/2), complete encapsulation and absence of tumor invasion) and 'poor characteristics' (which include tumor size ≥5 cm, presence of vascular invasion, higher stage (stage 3/4), higher tumor grade (grade 3/4), absence or incomplete encapsulation, presence of tumor invasion) (Figure S1). Normalized intensity for each lncRNAs between the poor clinical characteristics and good clinical characteristics for each clinical phenotype were assessed with Student t test using Partek Genomics Suite (Partek lnc., USA). LncRNAs with absolute fold change ≥1.5 and unadjusted P value < 0.05 are considered as significant. Univariate cox regression was applied to analyze association of lncRNAs with overall survival using Partek Genomics Suite (Partek lnc., USA). LncRNAs with hazard ratio (HR) > 1 or <1 with unadjusted P value <0.05 are considered as significant. Potentially oncogenic lncRNAs are defined as lncRNAs that are significantly higher in expression in both tumor compared to adjacent non-tumorous tissues and poor clinical characteristics versus good clinical characteristics or HR>1. On the other hand, potential tumor suppressive lncRNAs are defined as lncRNAs that are significantly lower in expression in both tumor compared to adjacent non-tumorous tissues and poor clinical characteristics compared to good clinical characteristics or HR<1.

### Validation in patient tissues using reverse transcription, real time quantitative polymerase chain reaction (RT-qPCR)

Validation of lncRNA was carried out on 61 pairs of HCC tumors tissues and adjacent non-tumorous tissues. Primer sequences are shown in Table S1. Reverse transcription of the extracted RNA was carried out using SuperScript^TM^ II Reverse transcriptase (Invitrogen, USA) and random primers (Invitrogen, USA), following manufacturer's instructions. RT-qPCR was performed using SYBR^TM^ Green PCR Master Mix (Life Technologies, UK) on 7500 Real-Time PCR System (Applied Biosystem, USA) as per instructions from the manufacturer. The expression levels of the transcripts were normalized to actin, a housekeeping gene and 2^-ΔΔCq^ was used to calculate relative expression 29. Wilcoxon signed-rank test was used to assess significance difference between LOC expression in HCC tumor tissues and adjacent non-tumorous tissues. Statistical significance is indicated if *P* value is < 0.05.

### Validation using Gene Expression Omnibus (GEO) datasets

Three HCC datasets were retrieved from GEO (http://www.ncbi.nlm.nih.gov/geo/) for validation. This includes GSE101728 (7 HCC paired tissues), GSE98269 (3 HCC paired tissues) and GSE115019 (12 HCC paired tissues). Differentially expressed LOC was identified by comparing expression of LOC in tumor tissues with adjacent non-tumorous tissues. Adjusted P values of <0.05 by Benjamin and Hochberg method and |logFC|>1 were considered as significantly different in expression.

### In silico analysis of LOC sequences

Information regarding protein coding potential and locus conservation of LOC sequences were obtained from Lncipedia 30-32 (Version 5.2) (https://lncipedia.org/). Conservation of LOC sequences in primate species was assessed using SyntDB 33 (http://syntdb.amu.edu.pl/).

### Cell culture

The immortalized (LO_2_) and transformed liver cells (HepG2 and Huh7) were maintained in High Glucose Dulbecco's Modified Eagle's medium (DMEM, Sigma Aldrich, USA), supplemented with 10% fetal bovine serum (FBS, Biological Industries, Isreal). LO_2_ cells was kindly donated by Professor Guan Xin Yuan, Director of Laboratory of Cancer Genetics, Hong Kong University while the HepG2 and Huh7 cells were obtained from ATCC. The cells were grown in a humidified 37 °C incubator with 5% CO_2_.

### Plasmid construction and preparation

LOC cDNA (synthesized by Bio Basic Inc. Ont, Canada) was cloned downstream of the human cytomegalovirus promoter using *BamH*1 and *Not*1 into pcDNA3.1+ plasmid (Thermo Scientific, USA). The plasmid was sequenced to ensure that the cDNA sequences are correct without mutation before large scale preparation of the plasmid using NucleoBond® Xtra Maxi EF kit (Macherey-Nagel, Germany) was performed.

### Cell transfection

To inhibit the expression of LOC in HepG2 cells, 500,000 cells were seeded in 6-well plate (Corning, USA) one day before transfection. A final concentration of 100 nM short interfering RNA (siRNA) targeting LOC is transfected into HepG2 cells using DharmaFECT 1 Transfection Reagents (Dharmacon, USA) following manufacturer's protocol. Both siRNAs targeting LOC (Lincode Human LOC101926913 siRNA- SMARTpool; siLOC) and control siRNA (ON-TARGETplus Lincode Non-targeting siRNA #1; siCtrl) were purchased from Dharmacon, USA. To overexpress LOC in LO_2_ cells, 250,000 cells were seeded a day before calcium phosphate transfection. On the day of transfection, the cells were first treated with 25 µM Chloroquine in 1 ml media to inhibit lysosomal degradation of DNA. A total volume of 150 µl calcium phosphate mixture was then prepared in a tube while vortexing in the following sequence: water, 4 µg pcDNA3.1+ plasmid (Thermo Scientific, USA) containing LOC insert, 9 µl of 2 M CaCl2, and 75 µl of 2X HEPES buffered saline. This mixture was added gently into the cells and incubated for eight hours before addition of 2 ml media. The transfected cells were harvested 36 hours post-transfection. pcDNA3.1+ plasmid (Thermo Scientific, USA) was used as a control (Ctrl) in overexpression. Total RNA was then extracted from the transfected cells using RNeasy Mini kit (Qiagen, Germany), following manufacturer's protocol.

### Cell proliferation assay

Trypan blue exclusion and automated real-time cell imaging (ARCI) were carried out to measure the proliferative characteristic of the transfected cells. Trypan blue exclusion was carried out by seeding transfected cells on 6-wells plate (Corning) and subsequent staining of the harvested cells at various time point (2nd, 4th, 6th, 8th day after seeding) using trypan blue. Cell counting was then carried out using the Invitrogen™ Countess II Automated Cell Counter (Thermo Fisher Scientific, USA). The ACRI system of IncuCyte™ ZOOM (Essen BioScience, USA), which automatically measures the relative cell confluency was employed to determine cell proliferation (three hours interval over 120 hours). The cell doubling time during the exponential growth phase was calculated using this formula: Doubling Time = duration * log(2)/[log(FinalConcentration)-log(InitialConcentration)].

### Anchorage independent growth

Anchorage independent growth was assessed using soft agar colony formation assay. 20,000 transfected LO_2_ cells or 25,000 transfected HepG2 cells were mixed with 0.6% UltraPure^TM^ Low Melting Point Agarose (Thermo Scientific, USA) in cell culture media and then seeded on top of 0.8% agarose layer. The cells were cultured for 30 days (LO_2_ cells) or 28 days (HepG2 cells) in a 37 °C incubator containing 5% CO_2_ with media replacement every 2-3 days. Colonies formed were then stained with 1% (w/v) methyl green in methanol for 15 minutes. Images of each well were taken on a light pad using Gel Doc^TM^ XR (Biorad, USA).

### Cell invasion assay

Cell invasion was measured using the Matrigel® Matrix Chamber (Corning, USA). 30,000 LO_2_ cells or 50,000 HepG2 cells were seeded on the matrigel layer together with medium containing 0.1% FBS. At the bottom of the transwell chamber, ten percent of FBS with medium was used as chemoattractant. The cells were then incubated for 96 hours. Cells on the upper surface of the chamber were removed using cotton swabs. On the other hand, the invaded cells were fixed using 3.7% formaldehyde, 100% methanol, KaryoMAX™ Giemsa Stain Solution (Invitrogen, USA) and Gurr solution for one minute each. Manual quantification of invaded cells based on four distinct fields were carried out (×40 magnification).

### Validation of successful overexpression and knockdown in cell lines using RT-qPCR/ reverse transcription, polymerase chain reaction (RT-PCR)

Successful knockdown of LOC in HepG2 cells was validated using RT-qPCR while successful overexpression of LOC in LO_2_ cells was validated using RT-PCR, which involved reverse transcription of extracted RNA from the cells lines, followed by cDNA amplification using HotStar Taq DNA polymerase (Qiagen), according to manufacturer's protocol. Primer sequences are shown in Table S1. After PCR amplification, the PCR products were separated on a 1% (w/v) agarose gel.

### RNA subcellular localization

1,000,000 Huh7 cells were harvested a day after seeding on 6-well plate. Cytoplasmic and nuclear RNA was extracted using Cytoplasmic and Nuclear RNA purification kit (Norgen Biotek, Canada), according to manufacturer's instruction. Expression of lncRNA targets in the cytoplasmic/nuclear fraction was assessed using RT-PCR followed by quantification of bands on gel image with ImageJ software 34. HOTAIR and actin were determined to indicate the successful extraction of nuclear and cytoplasmic RNAs respectively. The primer sequences for PCR amplification of HOTAIR and actin is shown in Table S1. Prediction of LOC subcellular localization was carried out using Lnclocator (http://www.csbio.sjtu.edu.cn/bioinf/lncLocator/) 35.

### RNA sequencing of LOC overexpressing and knockdown cells

Firstly, the quality of extracted RNA was assessed using Nanodrop, Agarose gel electrophoresis and Agilent 2100. Purification of mRNA was then performed using poly-T-oligo-attached magnetic beads before random fragmentation. Subsequently, the first cDNA strand was synthesized using random hexamers and M-MuLV reverse transcriptase. Following, the second cDNA strand was synthesized using DNA Polymerase I after RNase H treatment. After converting overhangs into blunt ends and adenylation of 3' ends, the cDNAs are ligated with sequencing adaptors. Selection of fragments of 150-200 bp length was then performed using AMPure XP system (Beckman Coulter, USA), followed by PCR amplification and purification of PCR products using AMPure XP beads. Quality of the purified products was checked using Qubit 2.0, Agilent 2100 and Q-PCR. Then, sequencing of the libraries was performed in illumina machines. After sequencing, raw image data files were transformed into sequencing reads by CASAVA base recognition (Base Calling). Low quality reads or reads containing adaptors were filtered before mapping the clean raw reads to human reference genome using STAR software. Finally, readcount of each gene was adjusted by TMM and differential analysis was carried out by EdgeR R package.

### Gene ontology and pathway analysis

ConsensusPathDB 36, 37 software was employed for Gene Ontology and Pathway analyses based on Reactome 38 and the Kyoto Encyclopedia of Genes and Genomes (KEGG) 39 databases. *P-*value <0.01 was used as a threshold to identify significantly enriched pathways.

### Statistical analyses

Experimental data is presented as mean ± standard error of the mean of three independent experiments. Statistical significance is indicated if *P* value < 0.05 based on paired Student's t-test which compares the difference between the experimental group and the control.

## Results

### Differential lncRNA expression profile in HCC

As previously reported 28, 1500 lncRNAs were found to be differentially expressed between the tumor (T) and adjacent non-tumorous (NT) liver in 49 HCC patients. 64.2% of these differentially expressed lncRNAs were intergenic, while 35.8% were genic (<3,000bp from protein coding genes), including 7.27% bidirectional, 2.0% exon-sense overlapping, 11.6% natural antisense, 4.0% intron sense-overlapping and 10.9% intronic antisense (Figure 1B) based on the reported genomic location classification 40.

### Association of deregulated lncRNAs with clinicopathological features

To investigate the clinical significance of the 1500 differentially expressed lncRNAs, we divided the clinical characteristics of the 49 patients into five categories as described previously 28 - tumor properties, tumor grade, tumor capsule, tumor invasion and overall survival status. (Figure S1). Among the 1500 differentially expressed lncRNAs, 480 of them were considered as clinically relevant as their expression was significantly deregulated in HCC patients and significantly associated with clinical characteristics in at least one clinical category (Figure 1C). 131 of these were potentially oncogenic lncRNAs as their expression was high in tumors and associated with worse prognosis. On the other hand, 349 of these were potentially tumor suppressing as their expression was low in tumors and associated with worse prognosis. Seven and 21 lncRNAs were found to be significantly up-and down- regulated (FDR<0.05, |Fold change|>2), respectively, and associated with worse prognosis of at least 3 clinical characteristics (Table S2) and these represent potential promising clinically relevant prognostic lncRNAs.

### LOC is overexpressed in the tumors of HCC patients and is significantly associated with cancer stage, tumor and vascular invasion as well as overall survival

**Figure 2:**
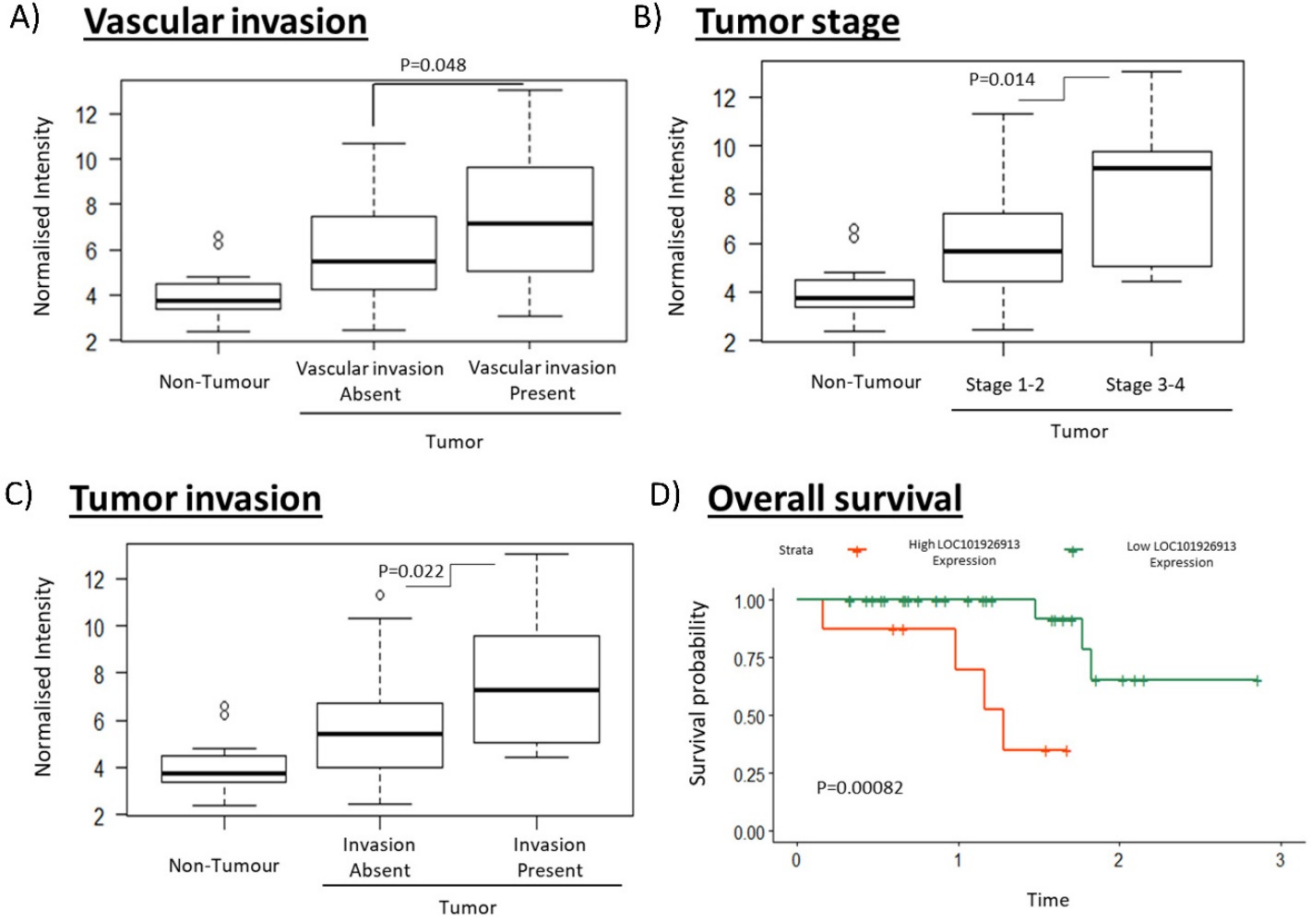
Clinical association of LOC in HCC patients including vascularization, cancer stage, tumor invasion and overall survival. **A-C)** The boxplots show expression of LOC in adjacent non-tumorous tissues, tumors associated with better clinical characteristics (absence of vascular invasion, stage 1-2, absence of tumor invasion) and tumors associated with worse clinical characteristics (presence of vascular invasion, stage 3-4, presence of tumor invasion). **D)** Survival curves shows that high LOC expression is associated with worse overall survival.

To identify clinically relevant lncRNAs that play important role in tumorigenesis, we selected a lncRNA for further characterization that have both the highest fold change values and is associated with the most number of clinical characteristics (Table S2). lncRNA LOC (LOC101926913; Seqname: NR_110185) (Table S2, boxed) was identified as a promising clinically relevant onco-lncRNA as it is associated with the most number of clinical phenotypes and is the most highly differentially expressed (7.09 fold) amongst those which are associated with the most number of clinical features (Table S2). High LOC expression was significantly associated with the presence of vascular invasion, higher tumor stage and the presence of tumor invasion (Figure 2A-C). Notably, high LOC expression in HCC patients was also associated with poorer overall survival (Figure 2D). These results suggest that LOC is a potential oncogenic lncRNA in HCC.

### Validation of expression in HCC patients and cell lines

**Figure 3:**
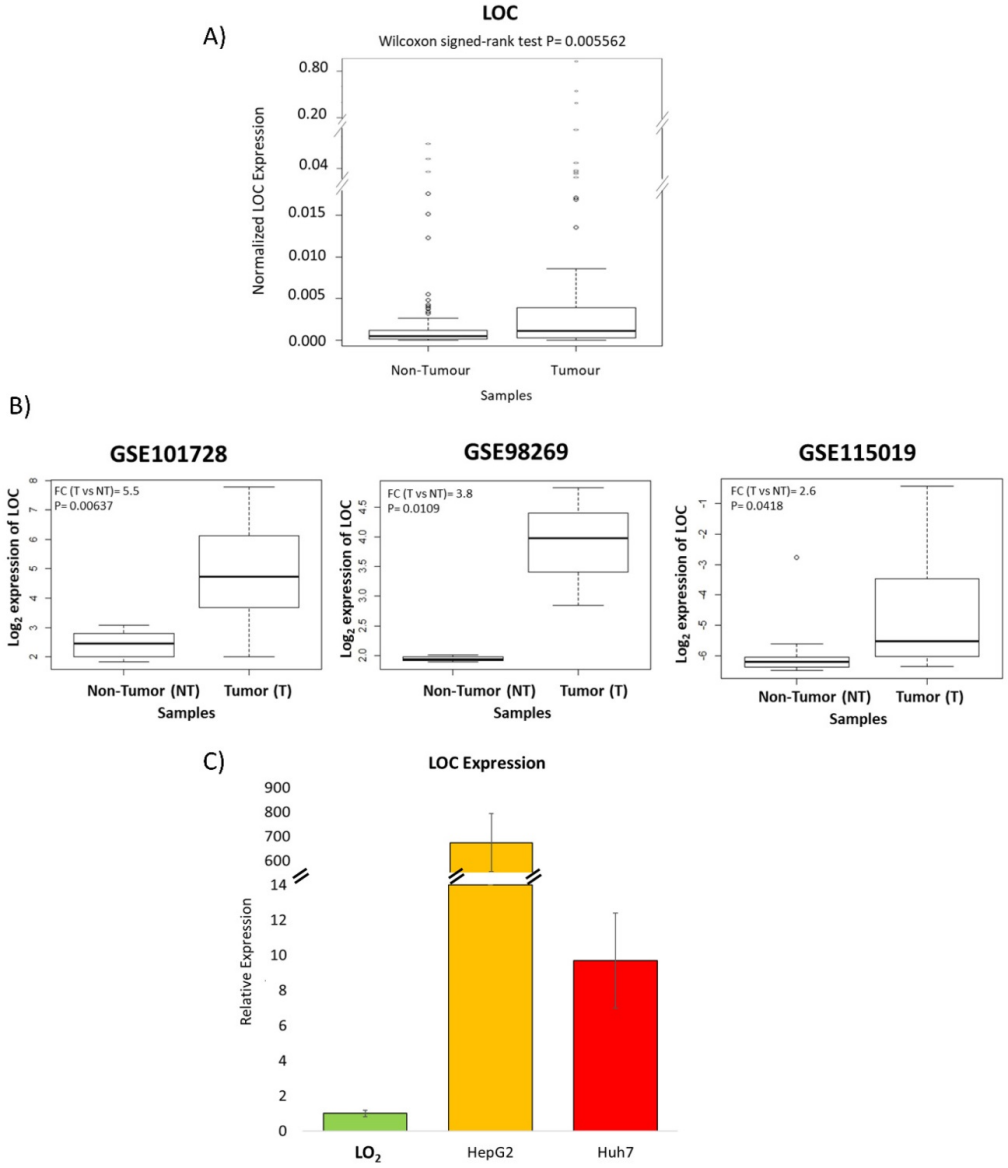
Validation of LOC expression in HCC patients and cell lines. **A)** Boxplot shows qPCR result of LOC expression in adjacent non-tumorous tissues and tumor tissues. **B)** Boxplot shows LOC expression in adjacent non-tumorous tissues and HCC tumor tissues in three GEO dataset (GSE101728, GSE98269 and GSE115019). **C)** Relative LOC expression in LO_2_ cells, HepG2 cells and Huh7 cells, measured by qPCR. The expression is normalized against actin. Data is presented as Mean±SE from three independent experiments.

RT-qPCR of tissues from 61 HCC patients was performed to validate the microarray observations that LOC is indeed over-expressed in the tumors of HCC patients. Figure 3A shows that expression of LOC was indeed significantly higher in HCC T compared to NT (P<0.01) using RT-qPCR consistent with observations from the microarray. Interrogation of the expression of LOC in the tumors of HCC patients in other populations in three additional public datasets (GSE101728, GSE98269 and GSE115019) revealed that LOC is indeed significantly over-expressed in the tumors of HCC patients (Figure 3B) consistent with observations in our population. Notably, LOC expression was also observed to be expressed in higher levels in transformed liver cell lines (HepG2 and Huh7) as compared to the immortal liver cell line (LO_2_) (Figure 3C).

### LOC is a novel lncRNA with no protein coding potential and its sequence is partially conserved in primates

As LOC is a novel lncRNA that has not been previously reported in the literature, *in silico* analyses was performed to better understand the LOC transcript. LOC is a 588bp long lncRNA that is transcribed from chromosome 2:171,556,878-171,627,276 (GRCh37/hg19)/ chromosome 2:170,700,368-170,770,773 (GRCh38/hg38), overlapping with 1 lncRNA (LINC01124) and 2 genes (SP5 and ERICH2) (Figure S2A). LOC is predicted not to have protein-coding potential from 5 different assessment tools (Figure S2B). While the LOC locus was not found to be conserved in some species including mouse, zebrafish, chimpanzee and flies in LNCipedia 30-32) its sequence was assessed by SyntDB 33 to be conserved in several primate species including chimpanzee but excluding Bonobo (Figure S2C).

### LOC promotes cell proliferation *in vitro*

**Figure 4:**
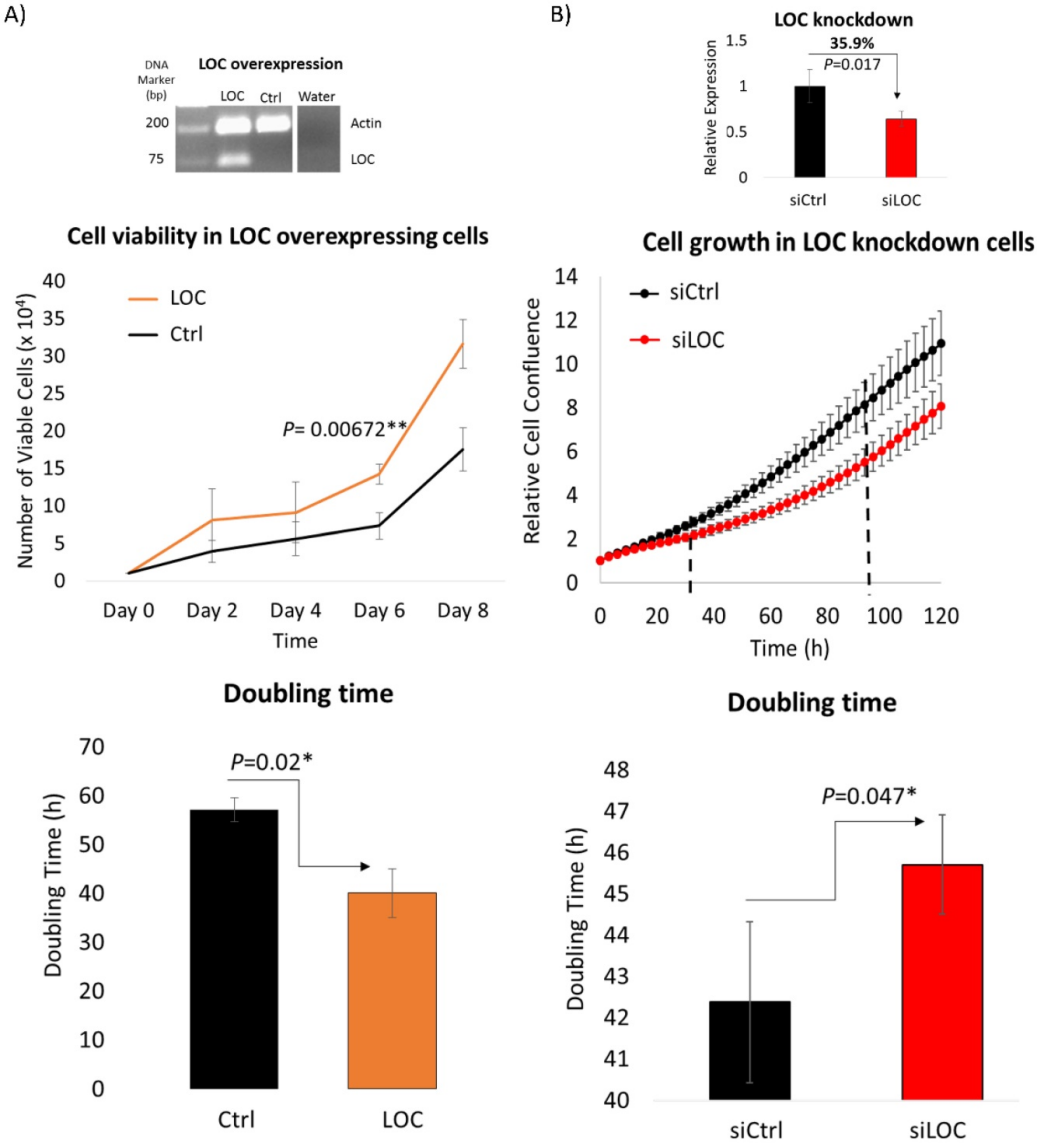
LOC enhances cell proliferation. **A)** (Top panel) Representative gel image shows multiplex PCR result of actin and LOC expression in LOC-overexpressing cells (LOC), control cells (Ctrl) and water as a negative control. (Middle panel) Cell proliferation profile of LOC-overexpressing cells (LOC) and control (Ctrl), measured by trypan blue exclusion cell counting method. (Bottom panel) The corresponding doubling time from day two till day eight is shown in the bar chart. **B)** (Top panel) Relative expression of LOC after knockdown in HepG2 cells (siLOC) compared to the knockdown control (siCtrl), as detected by qPCR. The expression is normalized against actin. (Middle panel) Cell growth result of LOC knockdown in HepG2 cells (siLOC) compared to control (siCtrl), measured with live cell imaging method. (Bottom panel) The corresponding doubling time from 36 hr till 108 hr is shown in the bar chart. All data are shown as mean±SE of three biological replicates. *:*P*<0.05 compared with control.

As lncRNA LOC is a novel, uncharacterized lncRNA, gain and loss-of-function experiments were performed to evaluate the role of lncRNA LOC in modulating cancer phenotypes. The first fundamental hallmark of cancer 41 examined was the ability of LOC to modulate cell proliferation using trypan blue exclusion cell counting and live cell imaging methods. Trypan blue exclusion cell counting method revealed that LOC-overexpressing cells enhanced cell proliferation with greater number of viable cells compared to controls and shorter doubling time (Figure 4A). Similarly, live cell imaging showed that inhibiting LOC expression with siRNAs lead to cells growing slower with greater doubling time (Figure 4B). Hence, LOC promotes cell proliferation.

### LOC enhances anchorage independent growth *in vitro*

**Figure 5:**
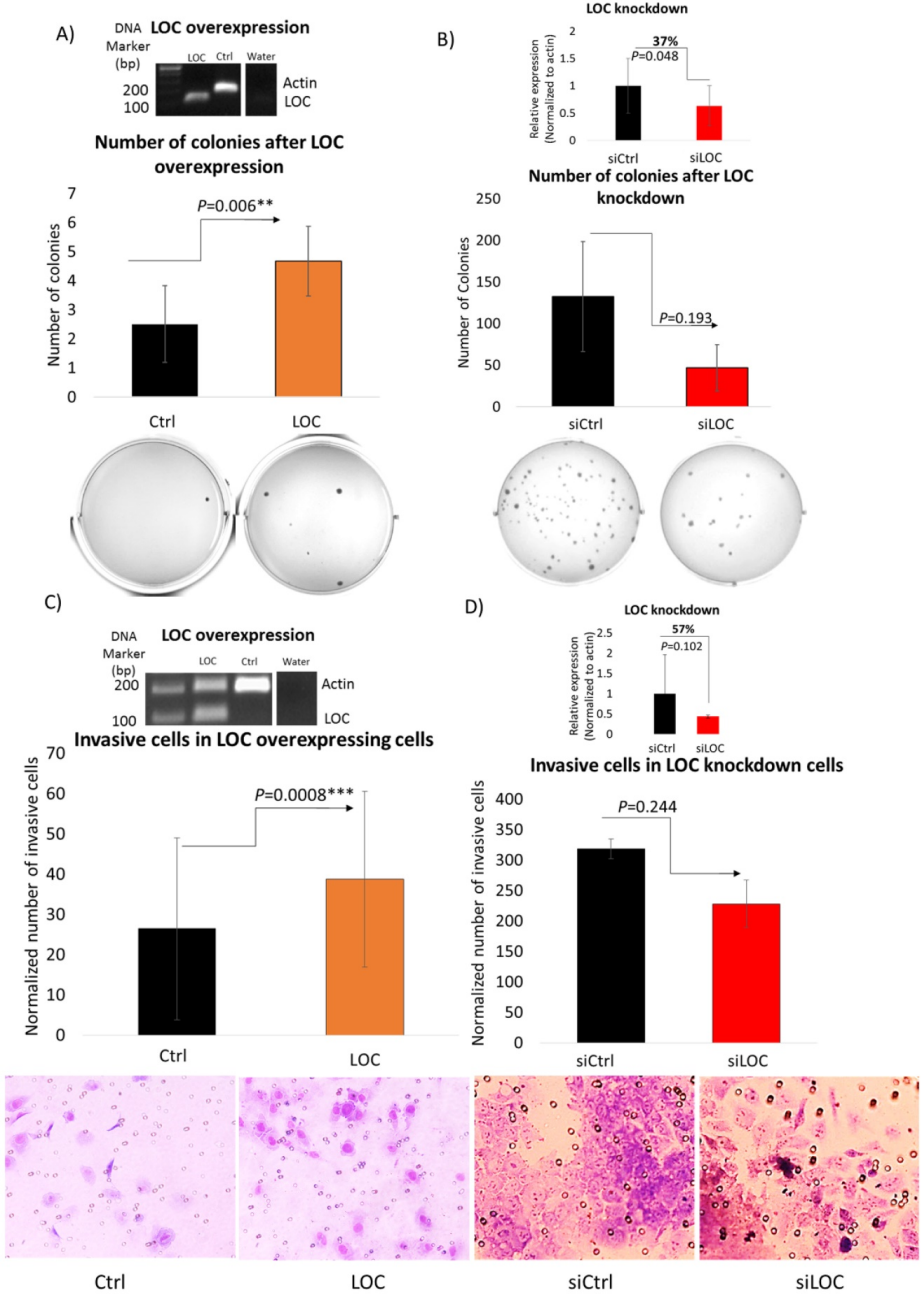
LOC promotes anchorage independent growth and cell invasive ability. **A)** (Top panel) Representative gel image shows multiplex PCR result of actin and LOC expression in LOC-overexpressing cells (LOC), control cells (Ctrl) and water as a negative control. (Middle panel) Number of colonies of LOC-overexpressing cells (LOC) and control (Ctrl) in soft agar. Colonies were stained with 1% (w/v) methyl green in methanol after incubating for 30 days post seeding. (Bottom panel) Representative figures taken from soft agar plates. **B)** (Top panel) Relative expression of LOC after knockdown in HepG2 cells (siLOC) compared to the knockdown control (siCtrl), as detected by qPCR. The expression is normalized against actin. (Middle panel) Number of colonies of LOC knockdown cells (siLOC) and control (siCtrl) in soft agar. Colonies were stained with 1%(w/v) methyl green in methanol after incubating for 28 days post seeding. (Bottom panel) Representative figures taken from soft agar plates. All data are shown as mean±SE of three biological replicates. **: *P*<0.01 compared with control. **C)** (Top panel) Representative gel image shows multiplex PCR result of actin and LOC expression in LOC-overexpressing cells (LOC), control cells (Ctrl) and water as a negative control. (Middle panel) Invasion profile of LOC-transfected cells (LOC) and control (Ctrl) using Transwell Matrigel assay. Cells were stained with Giemsa Stain solution before visualization under light microscope (x20 magnification). (Bottom panel) Representative pictures of invaded cells. ***: *P*<0.001 compared with control. **D)** (Top panel) Relative expression of LOC after knockdown in HepG2 cells (siLOC) compared to the knockdown control (siCtrl), as detected by qPCR. The expression is normalized against actin. (Middle panel) Invasion profile of LOC knockdown cells (siLOC) and control (siCtrl) using Transwell Matrigel assay. Cells were stained with Giemsa Stain solution before visualization under light microscope (x20 magnification). (Bottom panel) Representative pictures of invaded cells. All data are shown as mean±SE of three biological replicates. *:*P*<0.05 compared with control.

To evaluate the ability of LOC to modulate transformation through anchorage independent cell growth 42, soft agar assay was performed on LO_2_ cells with the LOC gene transfected and HepG2 cells with the siLOC introduced (Figure 5A and B). As evident in Figure 5A, LOC overexpression significantly enhanced the number of colonies formed on the soft agar (P<0.01). A reverse trend was observed in LOC-knockdown HepG2 cells, although it is not statistically significant (P=0.193) (Figure 5B). Hence, LOC likely alter transformation through anchorage independent growth suggesting that LOC may also modulate metastatic potential of tumors since anchorage independent growth signature was reported to be associated with metastasis 43.

### LOC increases cell invasive ability *in vitro*

As high expression of LOC was significantly associated with invasive tumors in HCC (Figure 2D) and cell invasion is an important factor in metastasis and cancer progression 44, we thus performed transwell matrigel invasion assay on LOC-overexpressing LO_2_ cells and LOC-knockdown HepG2 cells (Figure 5C and D) to evaluate LOC's ability to modulate cell invasion. As evident in Figure 5C, significantly higher number of invaded cells was observed in LOC-overexpressing LO_2_ cells compared to control. Conversely, fewer invaded cells were found in HepG2 cells carrying siLOC although it did not reach statistical significance (Figure 5D). Therefore, LOC may play a role in modulating cell invasive ability.

### LOC is preferentially localized in the cytoplasm

**Figure 6:**
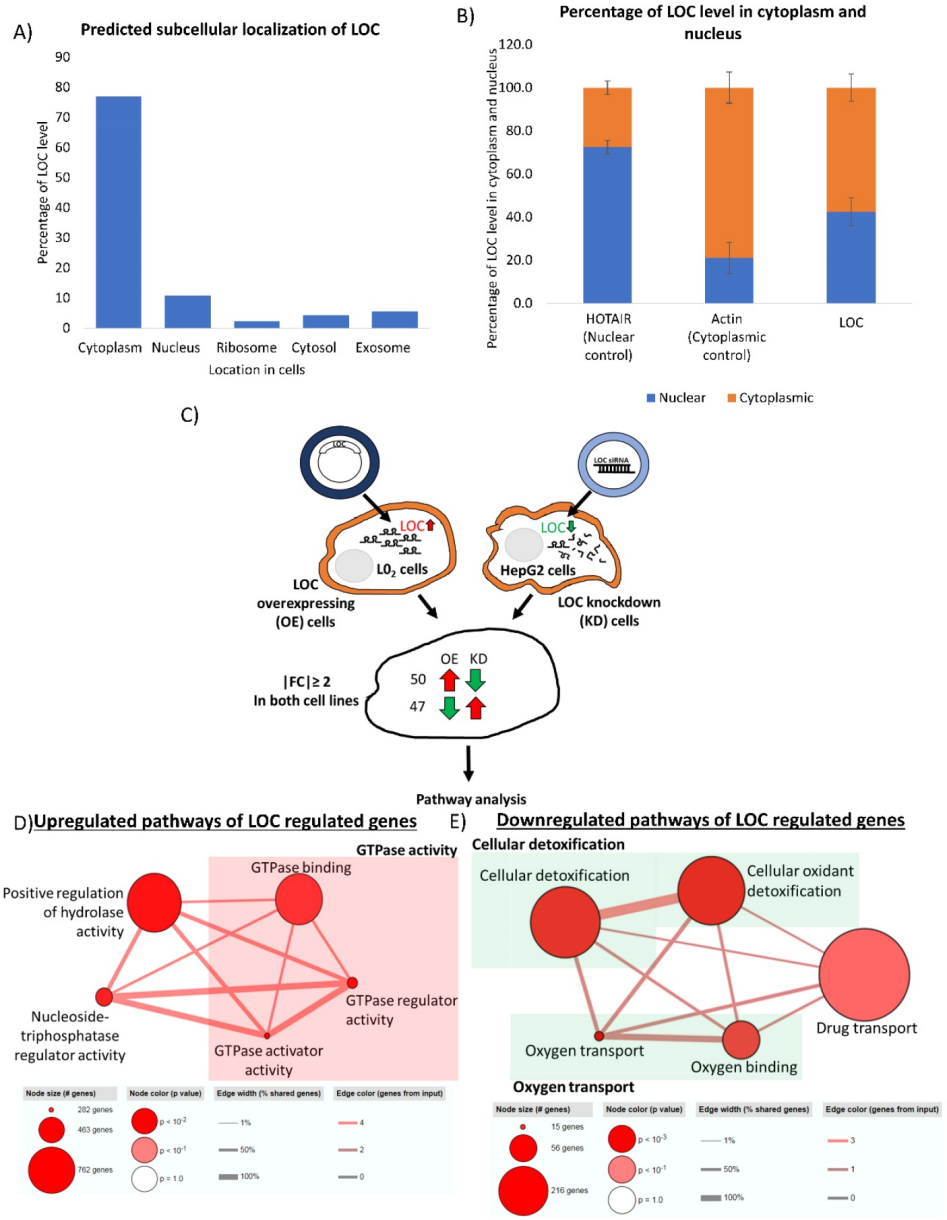
Identification of LOC regulated genes and their associated pathways. **A)** Subcellular localization of LOC is predicted using lnclocator (http://www.csbio.sjtu.edu.cn/bioinf/lncLocator/). **B)** Cytoplasmic and nuclear RNA fraction was separated using cytoplasmic and nuclear RNA purification kit and PCR was used to detect lncRNA expression. Quantification of lncRNA level is done by quantifying band intensity on gel image using Image J software. HOTAIR is a well characterized RNA localized in the nuclear while Actin is a well characterized RNA localized in the cytoplasm. Data are shown as mean±SE of three biological replicates. **C)** Workflow to identify potential genes regulated by LOC. RNA sequencing was performed in LOC overexpressing cells, LOC knockdown cells and respective controls. Genes that were expressed with |FC|≥ 2 in LOC vs Ctrl and siLOC vs siCtrl as well as expressed in opposite direction in both cells were included for pathway analysis. **D)** Pathways associated with LOC upregulated genes are GTPase activity, nucleoside-triphosphatase regulator activity, positive regulation of hydrolase activity. **E)** Pathways associated with LOC downregulated genes are cellular detoxification, oxygen transport and drug transport.

Location of RNAs provides insight to their potential interacting partners and hence their cellular role 45. Nuclear lncRNAs can interact with chromatin, regulate expression of genes at the transcriptional level and modulate RNA processing while cytoplasmic lncRNAs can affect mRNA translation, stability and modulate cellular signaling pathways 46. To gain insight into the potential function of this novel lncRNA, we first employed prediction tool from lnclocator to predict location of LOC in the cells 35. As shown in Figure 6A, nearly 80% of LOC is predicted to localize in cytoplasm. Subcellular fractionation revealed that LOC preferentially localized in the cytoplasm (cytoplasm:nucleus - 60:40) (Figure 6B) suggesting that LOC may play roles in modulating post-transcriptional regulation, translation or cellular signaling.

### LOC upregulates genes mainly involved in GTPase activities and downregulates genes involved in cellular detoxification as well as oxygen and drug transport

To glean some insights about the potential role of this onco-lncRNA, RNA sequencing was performed on LOC-overexpressing and LOC-knockdown cells and pathways analyses was performed on genes that are appropriately (up in one and down in the other or vice versa) deregulated in the two types of cell-lines (|Fold Change|≥2) (Figure 6C). Fifty genes were found to be upregulated in cells which overexpressed LOC but downregulated in cells with LOC-knockdown. These genes were found to be enriched in pathways associated with GTPase activity, positive regulation of hydrolase activity and nucleoside-triphosphatase regulator activity (Table S3A, Figure 6D). On the other hand, 47 genes that were downregulated in cells which overexpressed LOC but upregulated in cells with LOC-knockdown were mainly associated with cellular detoxification, oxygen transport and drug transport (Table S3B, Figure 6E). Two of these genes (HSD17B13 and FAM65C) were also found to be correlated with LOC expression in HCC patients (|PCC|≥0.6) (Table S3B_boxed in blue).

## Discussion

**Figure 7:**
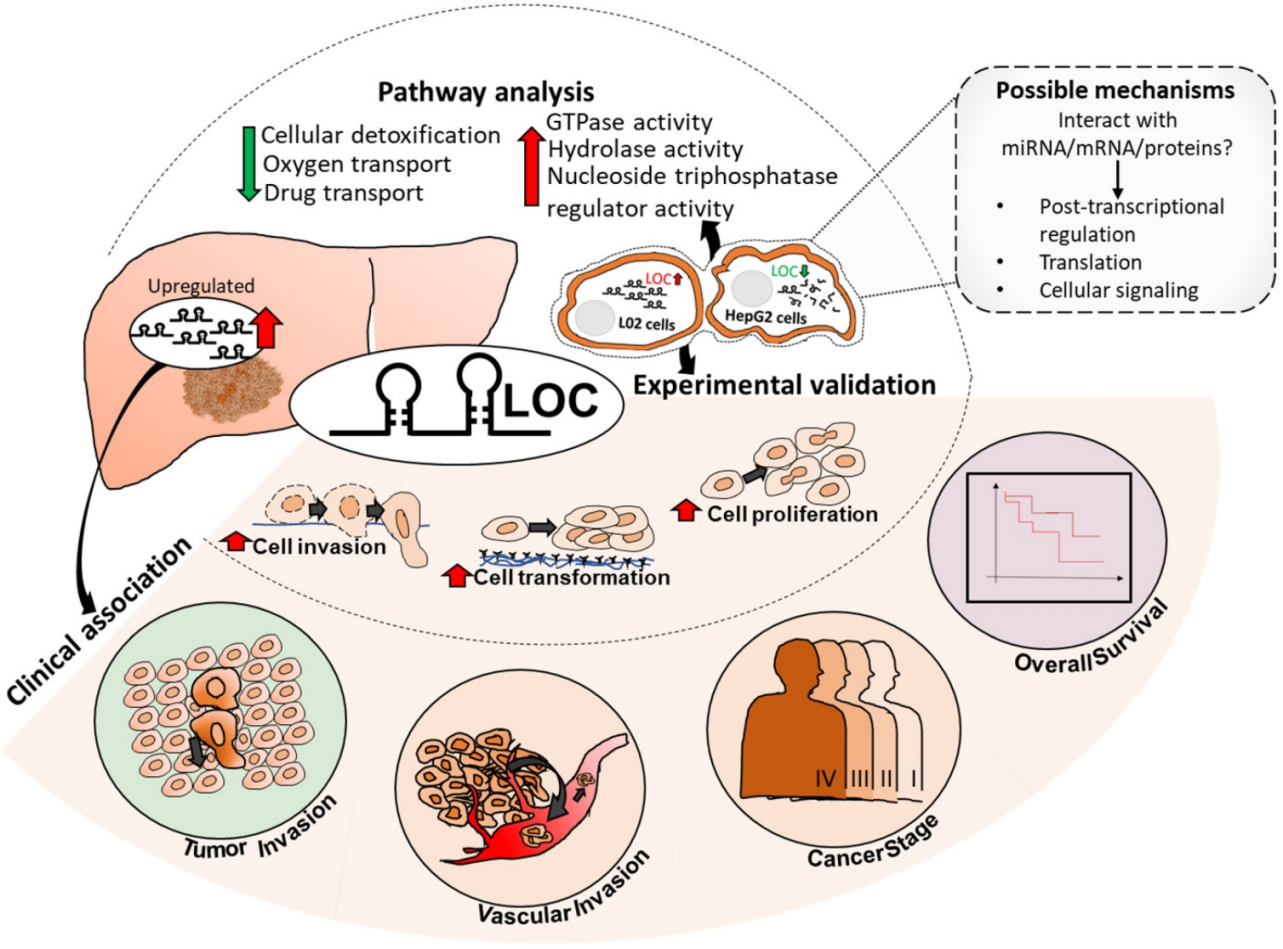
Summary of LOC expression, clinical association in patient tissues as well as experimental validated phenotypes and pathways. Possible mechanisms of LOC in gene regulation are shown in the dotted box with grey background. Red arrow: Upregulated; Green arrow: Downregulated.

As HCC is one of the most common causes of cancer mortality, identifying targets that modulate HCC prognosis may lead to therapeutic strategies for better patient outcome. LncRNAs represent promising therapeutic targets as they are potential regulators of genes and are able to function at RNA level without having to be translated, facilitating faster response after intracellular delivery 47. As lncRNAs usually exhibit tissue- or cancer-type specific expression 19, 48, there may be less likelihood of unintended side effects. Hence, this study aims to identify clinically relevant deregulated lncRNAs that may serve as potential prognostic targets by integrating lncRNA expression profiles with clinical characteristics association studies. Seven and 21 lncRNAs were found to be significantly up-and down-regulated (FDR<0.05, |Fold change|>2), respectively, as well as associated with worse prognosis of at least three clinical characteristics (Table S2). Of these, lncRNA LOC was selected for further characterization as it is associated with the most number of clinical phenotypes and is the most highly differentially expressed amongst those that are associated with the most number of clinical features (Table S2, boxed). The model presented in Figure 7 summarizes our findings of lncRNA LOC. Higher LOC expression was associated with higher tumor stage, tumor and vascular invasion as well as poorer overall survival (Figure 2B-E) suggesting that LOC may be a novel onco-lncRNA since there are no prior reports of this lncRNA and cancer.

Indeed its oncogenic potential was observed in gain- and loss-of function experiments where LOC was found to enhance cell proliferation (Figure 4), transformation (Figure 5A and B) and cellular invasion (Figure 5C and D), three important hallmarks of cancer 41. These experimental observations are consistent with the observation that higher lncRNA LOC expression is associated with clinical characteristics associated with poorer prognosis (Figure 2B-E). Higher tumor stage may be due to increased cell proliferation leading to increased tumor size as well as anchorage independent growth resulting in metastasis when LOC is over-expressed. The experimental observation that lncRNA LOC enhances cell invasion is indeed consistent with the clinical observation that high lncRNA LOC expression is associated with tumor invasion. Hence, high level of LOC may contribute to the worse clinical features observed in HCC patients.

Recent studies have reported that the act of transcription of lncRNAs is more likely to be functionally important than the actual RNA molecules 49-52. To get a further glimpse of potential function of this novel onco lncRNA -LOC or its locus, LOC locus and sequence conservation were preliminary investigated *in silico*. Locus of LOC was not conserved in other species while LOC exon sequences were found to be conserved in most of the primate species. These data suggests that RNA molecules may be important in its function which requires primary sequences conservation while transcription through LOC locus is less likely to be important 33. Hence, LOC is less likely to be involved in cis-regulation of its nearby genes in the nucleus 33, 53. This hypothesis is further supported by subcellular localization of LOC which also provides hints about possible regulatory role of LOC 45, 54. As evident in Figure 6A and B, LOC was found to preferentially localize in the cytoplasm, suggesting that it may play roles in post-transcriptional regulation, translation or cellular signaling perhaps through modulating the stability of mRNA or interacting with miRNAs or proteins 45, 55.

Notably, LOC was observed to up-regulate genes mainly involved in GTPase activity, positive regulation of hydrolase activity and nucleoside-triphosphatase regulator activity (Figure 6D). GTPases are known to mediate diverse cellular processes including transduction of cellular signals, regulation of cell division and cell differentiation, as well as protein production and translocation 56-61. Constitutive activation of G-proteins due to mutation was also reported to cause malignant transformation 62. Hence, LOC may upregulate GTPase activity to enhance cell transformation ability. Interestingly, among the LOC upregulated genes that are involved in GTPase activity (Table S3A), S100A9 gene was found to be overexpressed in HCC 63 and promote cell proliferation as well as invasion^64^, ^65^, which is consistent with our observations from functional assays in LOC-overexpressing/knockdown cells (Figure 4A and B, 5C and D). On the other hand, TAGAP gene was found to be correlated with lymphocyte infiltration in HCC, suggesting its upregulation may result in immune activation 66. It is thus worth investigating how LOC regulates these GTPase activity-related genes leading to the potential oncogenic effect in HCC.

Genes involved in cellular detoxification, oxygen and drug transport were found to be downregulated by LOC (Figure 6E). As oxidative stress is caused by the disruption of the balance between antioxidants and reactive oxygen species^67^, we hypothesize that LOC downregulate oxygen transport and detoxification process disrupting the balance leading to an increase of oxidative stress that was previously shown to affect gene expression and ultimately lead to HCC development 68, 69. Among the LOC downregulated genes that are involved in cellular detoxification (Table S3B), downregulation of LTC4S gene was also reported to associate with worse patient survival in HCC 70. As upregulation of LOC expression was also associated with worse patient survival, it is intriguing to study the mechanism of LOC in regulating LTC4S gene and patient survival. Two of the genes (HSD17B13 and FAM65C) that are down-regulated by LOC in *in vitro* experiments were also found to be negatively correlated with LOC in HCC patient tissues (|PCC|>0.6) (Table S3B_Boxed in blue). HSD17B13 has recently been reported as a liver restricted lipid droplet-associated protein whose expression is highly up-regulated in patients with non-alcoholic fatty liver disease although its physiological function remains unclear 71. Consistent with our observations, HSD17B13 was also reported to be downregulated in HCC 72, 73 and its low expression was associated with worse overall survival of HCC patients 72. However, its role in HCC remains unknown. Similarly, consistent with our finding, FAM65C was also reported to be downregulated in HCC although very little is known about this protein 74, 75. It is thus worthwhile to further characterize how lncRNA LOC deregulate the expression of HSD17B13 and FAM65C to modulate patient outcome.

In conclusion, we present a novel approach to identify clinically relevant lncRNAs that may modulate patient outcome. This novel strategy computationally integrates clinical association with differential lncRNA expression in HCC patients simultaneously to identify potential clinically relevant, differentially expressed lnRNAs that are more likely to play important roles in modulating the prognosis of HCC patients. This strategy is more time and resource efficient unlike previous reports which mainly focus on initially just identifying differentially expressed lncRNAs before experimental characterization followed by clinical association or vice versa. Using this strategy, we identified LOC as a potential clinically relevant, differentially expressed onco-lnRNA that modulates tumor-stage, vascular and tumor invasion and poorer overall survival of HCC patients (Figure 7). This is congruent with experimental observations that LOC expression in cells lead to enhanced proliferation, anchorage independent growth and invasion (Figure 7). LOC expression in cells up-regulated genes involved in GTPase activity, positive regulation of hydrolase activity and nucleoside-triphosphatase regulator activity while its expression down-regulated genes involved in cellular detoxification, oxygen and drug transport disrupting oxidative stress balance. Deeper characterization of the role of lncRNA LOC in modulating patient outcome is necessary before this onco-lncRNA can become a useful therapeutic target to improve patient outcome.

## Supplementary Material

Supplementary figures and tables.

## Abbreviations

HCC: Hepatocellular carcinoma
lncRNAs: long non-coding RNAs
ncRNAs: non-coding RNAs
DMEM: Dulbecco's Modified Eagle's medium
RT-qPCR: reverse transcription, real time quantitative polymerase chain reaction
Ct: Threshold Cycle
GEO: Gene Expression Omnibus
siRNA: Short interfering RNA
ARCI: automated real-time cell imaging
T: tumor tissues
NT: adjacent non-tumorous tissues
FDR: false discovery rate
LOC: LOC101926913
PCC: Pearson Correlation Coefficient
CNDP1: Carnosine dipeptidase 1
S100A9: S100 calcium-binding protein A9
TAGAP: T cell activation RhoGTPase activating protein
LTC4S: Leukotriene C4 synthase
HSD17B13: Hydroxysteroid 17-Beta Dehydrogenase 13
FAM65C: Family with Sequence Similarity 65 Member C
RASGEF1B: RasGEF Domain Family Member 1B
PLGLB2: Plasminogen Like B2
