# Supplementary material for: Highly deregulated lncRNA LOC is associated with overall worse prognosis in Hepatocellular Carcinoma patients
Source: J Cancer. 2021 Mar 30;12(11):3098–113. doi: 10.7150/jca.56340 (PMC8100808; doi:10.7150/jca.56340)
Supplement: Supplementary file 1 — Supplementary figures and tables. [file jcav12p3098s1.pdf]

# Supplemental Information

# Highly deregulated lncRNA LOC is associated with overall worse prognosis in Hepatocellular Carcinoma patients

Lee Jin Lim[1], Lay Hiang Ling[1], Yu Pei Neo[1,3], Alexander Y.F. Chung[2], Brian K.P. Goh[2], Pierce K.H. Chow[2,3,4] , Chung Yip Chan[2], Peng Chung Cheow[2], Ser Yee Lee[2], Tony K.H. Lim[5], Samuel S. Chong[7], London L.P.J.Ooi[2,3,4] and Caroline G. Lee[1, 3,6,8*]

*Corresponding author's email: bchleec@nus.edu.sg

**This file includes:**

**Figure S1** ........ 2

**Figure S2** ........ 3

**Table S1** ........ 4

**Table S2** ........ 5

**Table S3A** ........ 6

**Table S3B** ........ 9

# Figure S1

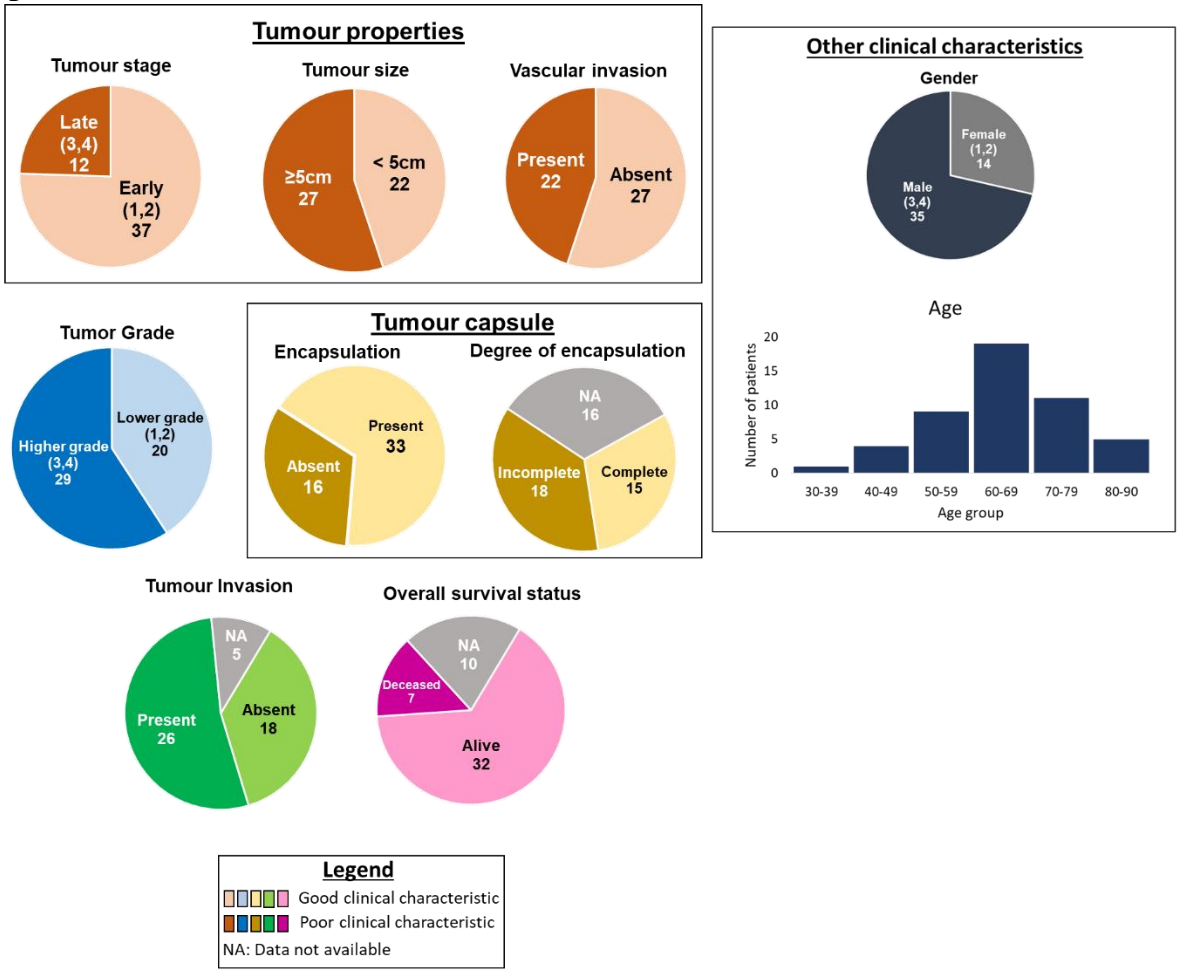


**Figure S1 Summary of clinical characteristics in 49 HCC patients**

Left panel: The eight clinical characteristics that are used for clincial analysis are further divided into 5 categories (Tumor properties, Tumor grade, Tumor capsule, Tumor invasion and Overall survival status). Within each clinical characteristics, it is further classified into good clinical characteristic and poor clinical characteristic. Dark orange/blue/yellow/green/purple background with white font: Poor clinical characteristic; Light orange/blue/yellow/green/purple background with black font: Good clinical characteristic. Grey background with white font: Data not available.

Right panel: Other clinical characteristics that are not included in the clinical analysis

# Figure S2

A)

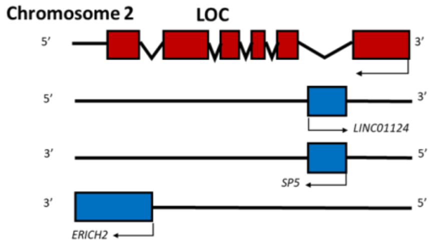


B)

**Assessment of protein coding potential of LOC**

| No. | Assessment tools | Raw result | Interpretation of raw result | Details of assessment tools |
|---|---|---|---|---|
| 1 | PRIDE reprocessing 2.0[a] | 0 | non-coding | Identification of peptides using mass spectrometry data |
| 2 | Lee translation initiation sites[b] | 0 | non-coding | Identification of translation initiation site using ribosome profiling |
| 3 | PhyloCSF score[c] | -130.5158 | non-coding | Identification of conserved coding region using multi-species nucleotide sequence alignment |
| 4 | CPAT coding probability[d] | 7.83% | non-coding | Assessment of coding gene features using logistic regression |
| 5 | Bazzini small ORFs[e] | 0 | non-coding | Identification of ribosome footprint throughout zebrafish embryogenesis using sequencing |

[a]Vizcaino, J.A, *et al.* 2016 update of the PRIDE database and its related tools. Nucleic Acids Res **2016**, 44, D447-456.
[b]Lee, S, *et al.* Global mapping of translation initiation sites in mammalian cells at single-nucleotide resolution. *Proc Natl Acad Sci U S A* **2012**, *109*, E2424-2432.
[c]Lin, M.F, *et al.* PhyloCSF: a comparative genomics method to distinguish protein coding and non-coding regions. *Bioinformatics* **2011**, *27*, i275-282.
[d]Wang, L ,*et al.* CPAT: Coding-Potential Assessment Tool using an alignment-free logistic regression model. *Nucleic Acids Res* **2013**, *41*, e74.
[e]Bazzini, A.A, *et al.* Identification of small ORFs in vertebrates using ribosome footprinting and evolutionary conservation. *EMBO J* **2014**, *33*, 981-993.

C)

**Orthologues of LOC in primate species**

| No. | Primate species | Orthologues | Transcript-transcript identity | Transcript-Genome identity |
|---|---|---|---|---|
| 1 | Chimpanzee | TCONS_00085165 | 32% | 5% |
| 2 | Bonobo | Not found | | |
| 3 | Gorilla | TCONS_00032815 | 0% | 1% |
| 4 | Orangutan | TCONS_00038588 | 0% | 3% |
| 5 | Rhesus | TCONS_00039370 | 0% | 2% |
| 6 | Macaca Fascicularis | TCONS_00121825 | 0% | 8% |
| 7 | Atys | TCONS_00096539 | 0% | 23% |
| 8 | Baboon | TCONS_00051038 | 0% | 8% |
| 9 | Green Monkey | TCONS_00034039 | 72% | 57% |
| 10 | Marmoset | TCONS_00081344 | 15% | 18% |
| 11 | Lemur | TCONS_00075553 | 0% | 0% |

**Figure S2 *In silico* analysis of LOC genomic location and sequence conservation**
A) Schematic diagram of LOC genomic location. Boxes in red: Exons of LOC; Boxes in blue: Exons of genes; Arrows: Transcription start site; Black lines: Introns
B) Protein coding potential of LOC using five assessment tools. This figure was adapted from Lncipedia (Version 5.2) (https://lncipedia.org/).
C) Orthologues of LOC in primate species. This figure shows the orthologues of LOC in 10/11 primate species (Adapted from SyntDB)(http://syntdb.amu.edu.pl/). Transcript-transcript identity: percentage of sequences that are aligned and identical between exons of LOC and exons of orthologoues; Transcript-genome identity: percentage of LOC that are aligned and identical to the syntenic genomic locus.

## Table S1 Primer sequences and annealing temperature used in this study

| Gene name | Forward primer sequences (5' to 3') | Reverse primer sequences (5' to 3') | Tm (°C) | Purpose |
|---|---|---|---|---|
| HOTAIR | GGTAGAAAAAGCAACCACGAAGC | ACATAAACCTCTGTCTGTGAGTGCC | 58 | PCR |
| Actin | ATGTTTGAGACCTTCACACC | AGGTAGTCAGTCAGGTCCCGGCC | 60 | PCR/qPCR |
| LOC101926913 | TCACTGTTGCTTCTCTGACCA | AGGGATTGGTGATAATCTTTTCTGA | 60 | PCR/qPCR |

# Table S2 Top 28 highly differentially expressed lncRNAs that have at least three clinical association.

| | LncRNA | | Expression in HCC | | | Clinical phenotypes | | | | | | | |
|---|---|---|---|---|---|---|---|---|---|---|---|---|---|
| | | | | | | Tumor properties | | | | Tumor capsule | | | |
| No. | seqname | GeneSymbol | p-value | FDR | Fold-Change | Tumor Stage | Tumor size | Vascular invasion | Tumor grade | Tumor capsule | Degree of encapsulation | Tumor invasion | Survival |
| 1 | NR_110185 | LOC101926913 | 2.11E-09 | 3.69E-08 | 7.09 | ↑ | | ↑ | | | | ↑ | ↑ |
| 2 | NR_038429 | RNASEH1-AS1 | 3.78E-13 | 3.72E-11 | 2.66 | ↑ | ↑ | ↑ | ↑ | | | | |
| 3 | T377450 | G089309 | 1.72E-05 | 8.58E-05 | 2.04 | ↑ | | | ↑ | ↑ | | | ↑ |
| 4 | NR_125797 | ZFPM2-AS1 | 5.71E-05 | 2.44E-04 | 3.57 | | | | ↑ | ↑ | | | ↑ |
| 5 | NR_046173 | LOC254896 | 1.63E-04 | 6.05E-04 | 2.67 | | ↑ | | ↑ | | | ↑ | |
| 6 | NR_024443 | LOC100133920 | 1.62E-10 | 4.37E-09 | 2.62 | | | ↑ | ↑* | ↑ | | | |
| 7 | T010075 | G002110 | 7.85E-07 | 5.93E-06 | 2.46 | | ↑ | ↑ | ↑ | | | | |
| 8 | T318968 | G074705 | 1.35E-02 | 2.78E-02 | -2.06 | ↓ | | ↓ | ↓ | | | ↓ | |
| 9 | T340880 | G079999 | 5.53E-11 | 1.84E-09 | -2.09 | ↓ | ↓ | | ↓ | ↓ | | | |
| 10 | NR_027300 | C3P1 | 1.86E-06 | 1.24E-05 | -2.27 | ↓ | ↓ | | ↓ | | | | ↓ |
| 11 | NR_024479 | FAM83A-AS1 | 3.51E-05 | 1.59E-04 | -2.71 | | ↓ | ↓ | ↓ | | | ↓ | |
| 12 | NR_046615 | ITIH4-AS1 | 2.05E-05 | 9.95E-05 | -2.00 | | ↓ | | ↓ | | | | ↓ |
| 13 | TCONS_00025782 | XLOC_012588 | 4.17E-04 | 1.38E-03 | -2.19 | | ↓ | | ↓ | ↓ | | | |
| 14 | T304533 | G071023 | 4.88E-06 | 2.85E-05 | -2.20 | ↓ | ↓ | | ↓ | | | | |
| 15 | T111422 | G026327 | 7.26E-10 | 1.50E-08 | -2.40 | | ↓ | | ↓ | | | ↓ | |
| 16 | NR_046612 | HTR2A-AS1 | 5.47E-10 | 1.19E-08 | -2.48 | | ↓ | | ↓ | | | ↓ | |
| 17 | ENST00000514791 | RP11-434D9.2 | 4.06E-09 | 6.50E-08 | -2.63 | | ↓ | ↓ | ↓ | | | | |
| 18 | ENST00000560903 | RP11-244F12.3 | 2.17E-10 | 5.56E-09 | -2.64 | ↓ | ↓ | | ↓ | | | | |
| 19 | uc002bxs.3 | DQ597539 | 1.05E-08 | 1.46E-07 | -2.78 | | ↓ | | ↓ | | | | ↓ |
| 20 | uc021trn.1 | DQ571055 | 8.44E-10 | 1.70E-08 | -2.79 | ↓ | | | ↓ | | | | ↓ |
| 21 | ENST00000608133 | RP11-523G9.3 | 5.05E-08 | 5.70E-07 | -3.55 | | | ↓ | ↓ | ↓ | | | |
| 22 | T099727 | G023548 | 5.68E-11 | 1.88E-09 | -3.83 | | | | ↓ | ↓ | | | ↓ |
| 23 | NR_125934 | LOC102723766 | 1.60E-10 | 4.35E-09 | -4.03 | | ↓ | | ↓ | ↓ | | | |
| 24 | ENST00000424701 | RP11-298H24.1 | 1.98E-11 | 8.03E-10 | -4.47 | | ↓ | | ↓ | | | ↓ | |
| 25 | NR_002733 | DGCR5 | 2.13E-06 | 1.39E-05 | -4.61 | ↓ | ↓ | ↓ | | | | | |
| 26 | TCONS_00023434 | XLOC_011275 | 2.08E-07 | 1.92E-06 | -6.21 | | ↓ | ↓ | ↓ | | | | |
| 27 | T074342 | G017089 | 2.17E-12 | 1.38E-10 | -7.48 | ↓ | ↓ | | ↓ | | | | |
| 28 | T099335 | G023451 | 3.86E-11 | 1.37E-09 | -8.61 | | | ↓ | ↓ | | | ↓ | |

Up arrow with red background: Upregulated in tumor tissues compared to adjacent non-tumor tissues; Down arrow with green background: Downregulated in tumor tissues compared to adjacent non-tumor tissues.

# Table S3A Genes that are upregulated in cells which overexpressed LOC but downregulated in cells with LOC-knockdown

| No. | Gene information | | LOC overexpression | | | LOC knockdown | | | Correlation of LOC with gene in patient tissues | Pathways/Gene enrichment | | | | |
|---|---|---|---|---|---|---|---|---|---|---|---|---|---|---|
| | | | Expression | | FC | Expression | | FC | | GTPase activity | | | Nucleoside-triphosphatase regulator activity | Positive regulation of hydrolase activity |
| | Seqname | GeneName | LOC | Ctrl | | siLOC | siCtrl | | PCC | GTPase binding | GTPase activator activity | GTPase regulator activity | | |
| 1 | ENSG00000185304 | RGPD2 | 0.24 | 0.05 | 4.58 | 0.00 | 0.10 | -16.81 | | ■ | ■ | ■ | ■ | ■ |
| 2 | ENSG00000164691 | TAGAP | 0.36 | 0.14 | 2.47 | 0.05 | 0.14 | -2.72 | | ■ | ■ | ■ | ■ | ■ |
| 3 | ENSG00000145087 | STXBP5L | 0.24 | 0.09 | 2.44 | 0.10 | 0.24 | -2.36 | | ■ | ■ | ■ | ■ | ■ |
| 4 | ENSG00000167037 | SGSM1 | 1.44 | 0.66 | 2.17 | 0.15 | 0.38 | -2.54 | | ■ | ■ | ■ | ■ | ■ |
| 5 | ENSG00000108961 | RANGRF | 0.12 | 0.00 | 19.16 | 0.00 | 0.14 | -24.72 | | ■ | | | | |
| 6 | ENSG00000074966 | TXK | 0.60 | 0.28 | 2.09 | 0.00 | 0.10 | -16.81 | | | | | | ■ |
| 7 | ENSG00000163220 | S100A9 | 0.30 | 0.14 | 2.07 | 0.05 | 0.14 | -2.72 | | | | | | ■ |
| 8 | ENSG00000204475 | NCR3 | 0.36 | 0.00 | 55.49 | 0.05 | 0.29 | -5.33 | | | | | | |
| 9 | ENSG00000197272 | IL27 | 0.18 | 0.00 | 28.24 | 0.00 | 0.19 | -32.63 | | | | | | |
| 10 | ENSG00000267849 | AL133262.1 | 0.18 | 0.00 | 28.24 | 0.00 | 0.10 | -16.81 | | | | | | |
| 11 | ENSG00000256591 | RP11-286N22.8 | 0.18 | 0.00 | 28.24 | 0.00 | 0.10 | -16.81 | | | | | | |
| 12 | ENSG00000172425 | TTC36 | 0.18 | 0.00 | 28.24 | 0.05 | 0.43 | -7.93 | -0.63 | | | | | |
| 13 | ENSG00000203546 | RP11-176H8.1 | 0.18 | 0.00 | 28.24 | 0.10 | 0.24 | -2.36 | | | | | | |
| 14 | ENSG00000157322 | CLEC18A | 0.18 | 0.00 | 28.24 | 0.19 | 0.43 | -2.16 | | | | | | |
| 15 | ENSG00000268098 | AP000688.1 | 0.12 | 0.00 | 19.16 | 0.00 | 0.10 | -16.81 | | | | | | |

| | | | | | | | | |
|---|---|---|---|---|---|---|---|---|
| 16 | ENSG00000126860 | EVI2A | 0.12 | 0.00 | 19.16 | 0.00 | 0.10 | -16.81 |
| 17 | ENSG00000185760 | KCNQ5 | 0.12 | 0.00 | 19.16 | 0.05 | 0.24 | -4.46 |
| 18 | ENSG00000149403 | GRIK4 | 0.12 | 0.00 | 19.16 | 0.29 | 0.76 | -2.57 |
| 19 | ENSG00000147003 | TMEM27 | 0.36 | 0.05 | 6.81 | 0.10 | 0.24 | -2.36 |
| 20 | ENSG00000267710 | AC006116.20 | 0.24 | 0.05 | 4.58 | 0.00 | 0.29 | -48.44 |
| 21 | ENSG00000242950 | ERVW-1 | 0.24 | 0.05 | 4.58 | 0.05 | 0.19 | -3.59 |
| 22 | ENSG00000159409 | CELF3 | 0.18 | 0.05 | 3.47 | 0.00 | 0.10 | -16.81 |
| 23 | ENSG00000082074 | FYB | 0.18 | 0.05 | 3.47 | 0.00 | 0.10 | -16.81 |
| 24 | ENSG00000187753 | C9orf153 | 0.18 | 0.05 | 3.47 | 0.15 | 0.33 | -2.23 |
| 25 | ENSG00000186952 | TMEM232 | 0.48 | 0.14 | 3.28 | 0.24 | 0.52 | -2.12 |
| 26 | ENSG00000163632 | C3orf49 | 0.30 | 0.09 | 3.03 | 0.05 | 0.29 | -5.33 |
| 27 | ENSG00000203867 | RBM20 | 1.44 | 0.47 | 3.02 | 0.00 | 0.14 | -24.72 |
| 28 | ENSG00000119725 | ZNF410 | 0.84 | 0.28 | 2.92 | 0.24 | 0.62 | -2.50 |
| 29 | ENSG00000150636 | CCDC102B | 0.84 | 0.28 | 2.92 | 0.39 | 0.95 | -2.42 |
| 30 | ENSG00000124529 | HIST1H4B | 0.42 | 0.14 | 2.88 | 0.00 | 0.10 | -16.81 |
| 31 | ENSG00000183479 | TREX2 | 0.96 | 0.38 | 2.51 | 0.15 | 0.33 | -2.23 |
| 32 | ENSG00000165794 | SLC39A2 | 0.60 | 0.24 | 2.50 | 0.05 | 0.29 | -5.33 |
| 33 | ENSG00000163519 | TRAT1 | 0.36 | 0.14 | 2.47 | 0.19 | 0.52 | -2.64 |
| 34 | ENSG00000119608 | PROX2 | 0.36 | 0.14 | 2.47 | 0.24 | 0.52 | -2.12 |
| 35 | ENSG00000181781 | ODF3L2 | 0.24 | 0.09 | 2.44 | 0.15 | 0.71 | -4.73 |
| 36 | ENSG00000100505 | TRIM9 | 0.24 | 0.09 | 2.44 | 0.05 | 0.19 | -3.59 |
| 37 | ENSG00000237289 | CKMT1B | 1.62 | 0.66 | 2.44 | 0.29 | 0.67 | -2.25 |
| 38 | ENSG00000136250 | AOAH | 0.12 | 0.05 | 2.35 | 0.00 | 0.10 | -16.81 |
| 39 | ENSG00000164304 | CAGE1 | 0.12 | 0.05 | 2.35 | 0.05 | 0.24 | -4.46 |
| 40 | ENSG00000233024 | PKD1P1 | 0.12 | 0.05 | 2.35 | 0.05 | 0.14 | -2.72 |
| 41 | ENSG00000188620 | HMX3 | 0.66 | 0.28 | 2.30 | 0.00 | 0.10 | -16.81 |
| 42 | ENSG00000212670 | AL161915.1 | 0.66 | 0.28 | 2.30 | 0.10 | 0.62 | -6.04 |
| 43 | ENSG00000072952 | MRVI1 | 0.54 | 0.24 | 2.25 | 0.05 | 0.14 | -2.72 |
| 44 | ENSG00000178226 | PRSS36 | 3.60 | 1.61 | 2.24 | 0.44 | 1.05 | -2.37 |
| 45 | ENSG00000122711 | SPINK4 | 0.84 | 0.38 | 2.20 | 0.10 | 0.29 | -2.82 |
| 46 | ENSG00000162482 | AKR7A3 | 0.72 | 0.33 | 2.15 | 0.24 | 0.90 | -3.65 |
| 47 | ENSG00000196648 | GOLGA6L9 | 0.60 | 0.28 | 2.09 | 0.24 | 0.57 | -2.31 |
| 48 | ENSG00000183396 | TMEM89 | 0.30 | 0.14 | 2.07 | 0.00 | 0.14 | -24.72 |

| | | | | | | | | | | |
|---|---|---|---|---|---|---|---|---|---|---|
| 49 | ENSG00000258643 | BCL2L2-PABPN1 | 0.30 | 0.14 | 2.07 | 0.05 | 0.14 | -2.72 | | |
| 50 | ENSG00000189350 | FAM179A | 1.08 | 0.52 | 2.07 | 0.05 | 0.24 | -4.46 | | |

Table shows the expression and fold change (FC) of genes that are upregulated in LOC-overexpressing cells but downregulated in LOC-knockdown cells, pearson correlation coefficient (PCC) of each gene in HCC patient tissues as well as pathways involved by the genes. The black box indicates the pathways that are involved by each gene.

## Table S3B Genes that are downregulated in cells which overexpressed LOC but upregulated in cells with LOC-knockdown

| No. | Gene information | | LOC overexpression | | | LOC knockdown | | | Correlation of LOC with gene in patient tissues | Pathways/Gene enrichment | | | | |
|---|---|---|---|---|---|---|---|---|---|---|---|---|---|---|
| | | | | | | | | | | Cellular detoxification | | Oxygen transport | | |
| | | | Expression | | FC | Expression | | FC | | Cellular detoxification | Cellular oxidant detoxification | Oxygen transport | Oxygen binding | Drug transport |
| | Seqname | GeneName | LOC | Ctrl | | siLOC | siCtrl | | PCC | | | | | |
| 1 | ENSG00000042062 | FAM65C | 0.00 | 0.28 | -43.89 | 0.49 | 0.24 | 2.02 | -0.62 | | | | | |
| 2 | ENSG00000170509 | HSD17B13 | 0.00 | 0.33 | -51.04 | 0.10 | 0.00 | 17.19 | -0.66 | | | | | |
| 3 | ENSG00000213931 | HBE1 | 0.06 | 0.42 | -6.48 | 0.88 | 0.38 | 2.28 | | ■ | ■ | ■ | ■ | ■ |
| 4 | ENSG00000196565 | HBG2 | 0.00 | 0.19 | -29.60 | 0.49 | 0.05 | 9.20 | | ■ | ■ | ■ | ■ | ■ |
| 5 | ENSG00000213316 | LTC4S | 0.00 | 0.14 | -22.45 | 0.15 | 0.05 | 2.84 | | ■ | ■ | | | |
| 6 | ENSG00000112077 | RHAG | 0.06 | 0.14 | -2.23 | 0.34 | 0.10 | 3.43 | | | | | | ■ |
| 7 | ENSG00000171798 | KNDC1 | 0.30 | 0.61 | -2.02 | 0.24 | 0.00 | 41.48 | | | | | | |
| 8 | ENSG00000213901 | SLC23A3 | 0.48 | 0.99 | -2.05 | 1.12 | 0.43 | 2.59 | | | | | | |
| 9 | ENSG00000187492 | CDHR4 | 0.18 | 0.38 | -2.06 | 0.15 | 0.05 | 2.84 | | | | | | |
| 10 | ENSG00000116544 | DLGAP3 | 0.18 | 0.38 | -2.06 | 0.29 | 0.10 | 2.95 | | | | | | |
| 11 | ENSG00000237452 | AC074212.3 | 0.06 | 0.14 | -2.23 | 0.24 | 0.10 | 2.47 | | | | | | |
| 12 | ENSG00000120215 | MLANA | 0.06 | 0.14 | -2.23 | 0.15 | 0.05 | 2.84 | | | | | | |
| 13 | ENSG00000186583 | SPATC1 | 0.06 | 0.14 | -2.23 | 0.29 | 0.10 | 2.95 | | | | | | |
| 14 | ENSG00000177340 | AC024940.1 | 0.06 | 0.14 | -2.23 | 0.29 | 0.10 | 2.95 | | | | | | |
| 15 | ENSG00000103489 | XYLT1 | 0.06 | 0.14 | -2.23 | 0.29 | 0.05 | 5.57 | | | | | | |
| 16 | ENSG00000185668 | POU3F1 | 0.06 | 0.14 | -2.23 | 0.29 | 0.05 | 5.57 | | | | | | |
| 17 | ENSG00000165194 | PCDH19 | 0.06 | 0.14 | -2.23 | 0.10 | 0.00 | 17.19 | | | | | | |
| 18 | ENSG00000143502 | SUSD4 | 0.06 | 0.14 | -2.23 | 0.10 | 0.00 | 17.19 | | | | | | |
| 19 | ENSG00000158560 | DYNC1I1 | 0.60 | 1.37 | -2.27 | 0.19 | 0.05 | 3.75 | | | | | | |

| | | | | | | | | | | |
|---|---|---|---|---|---|---|---|---|---|---|
| 20 | ENSG00000154710 | RABGEF1 | 0.12 | 0.28 | -2.29 | 0.24 | 0.10 | 2.47 | | |
| 21 | ENSG00000171860 | C3AR1 | 0.12 | 0.28 | -2.29 | 0.15 | 0.05 | 2.84 | | |
| 22 | ENSG00000162426 | SLC45A1 | 0.12 | 0.28 | -2.29 | 0.19 | 0.05 | 3.75 | | |
| 23 | ENSG00000198576 | ARC | 0.18 | 0.42 | -2.31 | 0.34 | 0.05 | 6.47 | | |
| 24 | ENSG00000008128 | CDK11A | 0.18 | 0.47 | -2.57 | 0.78 | 0.19 | 4.00 | | |
| 25 | ENSG00000155530 | LRGUK | 0.24 | 0.71 | -2.90 | 0.19 | 0.05 | 3.75 | | |
| 26 | ENSG00000248592 | TMEM110-MUSTN1 | 0.06 | 0.19 | -2.94 | 0.34 | 0.14 | 2.33 | | |
| 27 | ENSG00000177494 | ZBED2 | 0.06 | 0.19 | -2.94 | 0.24 | 0.05 | 4.66 | | |
| 28 | ENSG00000227921 | AL353791.1 | 0.06 | 0.19 | -2.94 | 0.24 | 0.00 | 41.48 | | |
| 29 | ENSG00000104901 | DKKL1 | 0.06 | 0.19 | -2.94 | 0.29 | 0.00 | 49.57 | | |
| 30 | ENSG00000100027 | YPEL1 | 0.18 | 0.66 | -3.58 | 0.63 | 0.29 | 2.19 | | |
| 31 | ENSG00000153237 | CCDC148 | 0.06 | 0.24 | -3.64 | 0.54 | 0.24 | 2.22 | | |
| 32 | ENSG00000183153 | GJD3 | 0.12 | 0.52 | -4.16 | 0.49 | 0.19 | 2.51 | | |
| 33 | ENSG00000176732 | PFN4 | 0.18 | 0.80 | -4.34 | 0.15 | 0.00 | 25.28 | | |
| 34 | ENSG00000259224 | SLC35G6 | 0.06 | 0.28 | -4.35 | 0.15 | 0.00 | 25.28 | | |
| 35 | ENSG00000188389 | PDCD1 | 0.00 | 0.14 | -22.45 | 0.39 | 0.14 | 2.66 | | |
| 36 | ENSG00000186231 | KLHL32 | 0.00 | 0.14 | -22.45 | 0.15 | 0.05 | 2.84 | | |
| 37 | ENSG00000131771 | PPP1R1B | 0.00 | 0.14 | -22.45 | 0.10 | 0.00 | 17.19 | | |
| 38 | ENSG00000268941 | MGC4294 | 0.00 | 0.14 | -22.45 | 0.10 | 0.00 | 17.19 | | |
| 39 | ENSG00000153902 | LGI4 | 0.00 | 0.14 | -22.45 | 0.24 | 0.00 | 41.48 | | |
| 40 | ENSG00000105251 | SHD | 0.00 | 0.14 | -22.45 | 0.24 | 0.00 | 41.48 | | |
| 41 | ENSG00000137101 | CD72 | 0.00 | 0.19 | -29.60 | 0.10 | 0.00 | 17.19 | | |
| 42 | ENSG00000160951 | PTGER1 | 0.00 | 0.24 | -36.74 | 0.83 | 0.33 | 2.46 | | |
| 43 | ENSG00000102539 | MLNR | 0.00 | 0.24 | -36.74 | 0.54 | 0.14 | 3.64 | | |
| 44 | ENSG00000105261 | OVOL3 | 0.00 | 0.24 | -36.74 | 0.19 | 0.05 | 3.75 | | |
| 45 | ENSG00000171747 | LGALS4 | 0.00 | 0.28 | -43.89 | 0.29 | 0.05 | 5.57 | | |
| 46 | ENSG00000232671 | RP11-126K1.2 | 0.00 | 0.28 | -43.89 | 0.10 | 0.00 | 17.19 | | |
| 47 | ENSG00000189269 | C22orf43 | 0.00 | 0.47 | -72.48 | 0.29 | 0.10 | 2.95 | | |

Table shows the expression and fold change (FC) of genes that are downregulated in LOC-overexpressing cells but upregulated in LOC-knockdown cells, pearson correlation coefficient (PCC) of each gene in HCC patient tissues as well as pathways involved by the genes. The black box indicates the pathways that are involved by each gene. Blue box: LOC-downregulated genes that are correlated with LOC expression in patient tissues samples.
